# Exploratory transcriptomic profiling of melittin’s efficacy in the treatment of murine cervical cancer cells

**DOI:** 10.3389/fcell.2026.1975665

**Published:** 2026-10-14

**Authors:** Jianrong Jiang, Yuwei Zhang, Mengyi Wang, Ronghua Zhang, Yueliang Li, Jianfeng Qiu, Dafu Chen, Tizhen Yan, Rui Guo, Yanhui Liu

**Affiliations:** 1 Guangdong Medical University, Dongguan, China; 2 Dongguan Maternal and Children Health Hospital, Dongguan, China; 3 Apitherapy Research Institute of Fujian Agriculture and Forestry University, Fuzhou, China; 4 College of Bee Science, Fujian Agriculture and Forestry University, Fuzhou, China; 5 National & Local United Engineering Laboratory of Natural Biotoxin, Fuzhou, China; 6 Dongguan Technology Transfer Center of National & Local United Engineering Laboratory of Natural Biotoxin, Dongguan, China

**Keywords:** AP-1 complex, cervical cancer, melittin, transcriptomics, U14 cell

## Abstract

**Introduction:**

Melittin, a highly active antimicrobial peptide derived from honeybee venom, holds immense pharmacological potential against solid tumors, yet its specific anti-tumor efficacy and transcriptomic dynamics in cervical carcinoma remain inadequately characterized.

**Methods:**

This study systematically evaluated the anti-cancer properties and potential molecular mechanisms of melittin on murine U14 cervical cancer cells.

**Results:**

Phenotypic assessments demonstrated that melittin potently inhibited U14 cell viability and dose-dependently suppressed cellular migration, culminating in near-complete migratory arrest at high concentrations. Additionally, flow cytometry revealed a dramatic, dose-dependent surge in the Annexin V+/PI+ cell population. Given the well-established membranolytic properties of melittin, this lethal effect structurally corroborated by scanning electron microscopy (SEM) showing severe plasma membrane perforation and morphological collapse, may reflect a possible mixture of true late apoptosis and secondary necrosis, though the exact mode of cell death remains undefined. Following the rigorous identification of differentially expressed genes (DEGs) in U14 cells, subsequent enrichment analyses indicated that the physical membrane disruption inflicted by melittin translated into a severe metabolic crisis, marked by a global suppression of ribosomal biogenesis and mitochondrial oxidative phosphorylation. Furthermore, melittin significantly downregulated *Etv2*, a key transcription factor in vascular development, suggesting not only a potential suppression of angiogenesis-related networks but also the potential involvement of an AP-1 transcriptional stress complex (evidenced by the profound upregulation of *Jun*, *Fos*, and *Fosb*).

**Discussion:**

Collectively, these findings outline the pharmacological networks underlying melittin’s cytotoxicity, providing an exploratory molecular framework for its future development as a natural therapeutic agent against cervical cancer.

## 1 Introduction

Cervical cancer is a malignant tumor originating from the epithelial cells of the cervix (Schiffman et al., 2016). The cervix constitutes the lower portion of the uterus, connecting it to the vagina. Tumors form when cervical cells undergo abnormal DNA alterations and proliferate uncontrollably (Cohen et al., 2019). Globally, there are approximately 661,000 new cases of cervical cancer and 348,000 relevant deaths annually. In China, the incidence and mortality rates of cervical cancer rank among the highest worldwide, and the disease presents a trend toward younger demographics, posing a severe threat to women’s health and lives (Sung et al., 2026). Although the widespread administration of human papillomavirus (HPV) vaccines and advancements in screening technologies have significantly reduced the incidence of cervical cancer, it remains a leading cause of cancer-related mortality among women in developing countries due to limited medical resources and inadequate screening coverage (Canfell et al., 2020). Consequently, cervical cancer continues to be a pressing global public health challenge. Currently, the primary treatment modalities for early-stage cervical cancer include surgical resection, radiotherapy, and chemotherapy, whereas concurrent chemoradiotherapy or targeted therapy is employed for advanced or recurrent cases (Bhatla et al., 2025). However, traditional chemotherapeutic agents, such as cisplatin and paclitaxel, are associated with severe toxic side effects. Furthermore, tumor cells are prone to developing multidrug resistance, giving rise to treatment failure and disease progression (Kitagawa et al., 2015; Zhu et al., 2016). In recent years, despite the promise shown by immune checkpoint inhibitors (e.g., protein 1/programmed death-ligand 1 (PD-1/PD-L1) antibodies) for certain patients, the overall response rate remains below 20%, and the treatment costs are prohibitively high (Cohen et al., 2019; Chung et al., 2016). Hence, developing novel anti-cervical cancer drugs with high efficacy and low toxicity, as well as deeply elucidating their mechanisms of action, is of significant clinical and scientific value.

Melittin, constituting approximately 50% of the dry weight of bee venom, is its principal active component. As a cationic amphiphilic peptide composed of 26 amino acids, melittin is considered a highly potent candidate for cancer therapy due to its robust antitumor activity (Rady et al., 2017; Guha et al., 2021; Liu et al., 2016). Its antitumor mechanism relies not only on its membranolytic properties but also on its ability to effectively eliminate tumor cells by inducing necrosis and apoptosis (Ceremuga et al., 2020; Sun et al., 2025). To date, certain progress has been made in research regarding the anti-cervical cancer effects of melittin. Studies have shown that in cervical cancer models, melittin exhibits significant dose-dependent cytotoxicity, inducing G_0_/G_1_ cell cycle arrest and mitochondria-mediated apoptosis (Tipgomut et al., 2018). In addition, melittin can specifically downregulate the expression of the E6 and E7 oncoproteins of high-risk HPV types, such as papillomavirus 16 and human papillomavirus 18 (HPV16 and HPV18), thereby selectively killing HPV-positive cervical cancer cells (Kim et al., 2015). Nevertheless, current research on the efficacy of melittin against cervical cancer remains in its preliminary stages, and the underlying molecular regulatory networks require further in-depth elucidation.

In the present study, using the murine cervical cancer U14 cell line as a model, we integrated macroscopic phenotypic characterizations, including cell viability, flow cytometry-based apoptosis assays, wound healing, and ultrastructural evaluations via scanning electron microscopy (SEM) with global transcriptome sequencing (RNA-seq) to systematically screen differentially expressed genes (DEGs) following melittin treatment. While the raw transcriptomic dataset utilized in this study was previously generated and published (Zhang et al., 2026), the comprehensive macroscopic phenotypic characterizations and transcriptomic analyses are entirely novel. Specifically, our primary focus highlights melittin’s capacity to couple direct physical membrane disruption with a complete paralysis of ribosomal translation and oxidative phosphorylation, the downregulation of the *Etv2* transcription factor (suggesting a potential to suppress angiogenesis-related networks), and the profound upregulation of the activator protein 1 (AP-1) transcriptional stress interactome. This work aims to thoroughly elucidate the multi-targeted molecular mechanisms of melittin against cervical cancer at both the structural and transcriptional levels, thereby providing a foundation for its clinical translation and application in the future.

## 2 Materials and methods

### 2.1 Cell culture and melittin

Murine cervical cancer cells (Xiamen Immocell Biotechnology), maintained at the Laboratory of Host-Pathogen Interaction Mechanisms and Precision Medicine, College of Bee Science of Fujian Agriculture and Forestry University, were cultured in complete medium (Thermo Fisher Scientific, Waltham, MA, United States of America) at 37 °C in a 5% CO_2_ atmosphere. Melittin (purity ≥95%) was purchased from Selleck (United States of America).

### 2.2 Cell viability and colony formation assays, and dose selection

Following the instructions, cell proliferation and viability were assessed using the Cell Counting Kit-8 (CCK-8) assay (Beyotime, Shanghai, China). Briefly, U14 cells were seeded into 96-well plates, incubated overnight, and treated with varying concentrations of melittin (0, 2, 4, 6, and 8 μg/mL) for 20 min. OD was measured at 450 nm to determine the IC50. For the colony formation assay, cells were seeded into 6-well plates 500 cells/well, exposed to the same melittin gradient for 20 min, and cultured in drug-free medium for an additional 7 days. Macroscopic colonies were fixed with 4% paraformaldehyde and stained with 0.1% crystal violet. On basis of the fact that melittin drastically suppressed the colony-forming capacity of U14 cells in a dose-dependent manner, alongside the CCK-8 viability data, 4 μg/mL was rigorously selected as the optimal intervention concentration for all subsequent transcriptomic and mechanistic analyses.

### 2.3 Wound healing assay

U14 cells were seeded into six-well plates and grown to full confluence. An artificial wound was created by scratching the cell monolayer linearly with a sterile 200 μL pipette tip. After being washed with PBS to remove cellular debris, the cells were cultured in medium containing melittin (0, 2, 4, 6, and 8 μg/mL). Images of the identical wounded areas were captured at 0 h and 16 h post-wounding using inverted phase-contrast microscope (Sunny Optical, China).

### 2.4 Annexin V-FITC/PI apoptosis assay

U14 cells treated with varying concentrations of melittin (0, 2, 4, 6, and 8 μg/mL) were harvested and washed with cold PBS. The collected cells were resuspended in 1× binding buffer and double-stained with Annexin V-FITC and Propidium Iodide (PI) (Yeasen, Shanghai, China) in the dark according to the protocol. The stained cells were immediately acquired utilizing a flow cytometer, and the proportions of apoptotic cells across different sub-populations were quantified.

### 2.5 Transcriptome data source

The raw RNA-seq dataset utilized for bioinformatic reanalysis in this study was derived from our previously obtained transcriptome data (Zhang et al., 2026). As detailed previously, melittin- and un-treated U14 cell samples (with three independent biological replicates per group, n = 3) were subjected to total RNA extraction using TRIzol. RNA integrity was strictly evaluated, with all samples yielding an RNA Integrity Number (RIN) > 7.0. cDNA library construction and deep sequencing were performed on the Illumina NovaSeq platform (150 bp paired-end reads), achieving an average sequencing depth of approximately 38.2 million raw reads per sample. Raw data underwent strict quality control to remove adapter sequences, poly-N containing reads, and low-quality sequences (Phred score <20).

For downstream reanalyses in this work, the resulting high-quality clean reads were aligned to the murine reference genome (GRCm39) utilizing HISAT2 (version 2.2.1), and gene expression levels were quantified using StringTie (version 2.1.6). To ensure robust statistical downstream analysis, low-expression genes were strictly filtered out prior to differential analysis. This refined dataset was then subjected to the advanced, multi-level bioinformatics pipeline (GSEA, Reactome, PPI) to decode the cellular stress mechanisms.

### 2.6 SEM detection

U14 cells treated with 4 μg/mL melittin or complete medium for 20 min were evaluated for gross morphological alterations utilizing an inverted microscope. For ultrastructural imaging, cells were fixed in 2.5% glutaraldehyde at 4 °C overnight, post-fixed with 1% OsO_4_ for 1 h, and dehydrated through a graded ethanol series. Following critical-point drying and gold sputter-coating, the cell surface topography was visualized using a scanning electron microscope (Carl Zeiss, Oberkochen, Germany).

### 2.7 DEG screening and enrichment analysis

DEGs between the un- and melittin-treated groups were screened using the DESeq2 package (v1.22.2), based on the cutoff criteria of an absolute log_2_ fold change (|log_2_FC|) ≥ 1 and *Q* value <0.05. To decipher the biological implications of these transcriptomic alterations, GO enrichment was performed via the clusterProfiler package using a hypergeometric test.

To further map these DEGs into systemic biological pathways, we queried the KEGG (https://www.kegg.jp/) and Reactome (https://reactome.org/) databases, defining significance as *P* < 0.05. For GO and KEGG enrichment analyses, the background gene set was defined as all genes within the murine reference genome possessing corresponding functional annotations, and unannotated genes were excluded from the background to calculate precise *P* and *Q* values. Instead of relying solely on predefined DEGs, we also assessed global transcriptomic shifts using GSEA, with significant sets defined by an absolute normalized enrichment score (|NES|) > 1, nominal *P* value <0.05, and false discovery rate (FDR) < 0.25. Finally, to identify core regulatory hubs within the altered pathways, a protein-protein interaction (PPI) network was constructed utilizing STRING (v11.5) and visualized in Cytoscape (v3.9.1). Interactions with a combined confidence score >0.400 (medium confidence) were retained to ensure reliability.

### 2.8 qRT-PCR analysis

Total RNA was isolated from the cervical cancer cells utilizing the Accurate RNA Extraction Kit (Accurate Biotechnology). Following the assessment of RNA yield and integrity via NanoDrop spectrophotometry and agarose gel electrophoresis, equal amounts of purified RNA were subjected to reverse transcription using the Hifair® III cDNA Synthesis Kit (Yeasen, Shanghai, China). To validate the reliability of the used transcriptome data, six representative genes (*Fos*, *Fosb*, *Jun*, *Nr1i2*, *Prdm1*, and *Egr1*) were selected for quantitative PCR (qPCR), since *Fos*, *Fosb*, and *Jun* serve as central biological hubs driving the AP-1 transcriptional stress complex (Atsaves et al., 2019), while *Egr1*, *Nr1i2*, and *Prdm1* are critical regulators dynamically involved in early stress responses and transcriptomic reprogramming (Wang et al., 2021). Reaction was conducted on a QuantStudio three system (Applied Biosystems) using SYBR Green chemistry (Yeasen, Shanghai, China). The amplification was performed in a 20 μL reaction volume with an initial denaturation at 95 °C for 3 min, followed by 40 cycles of 95 °C for 15 s, 58 °C for 30 s, and 72 °C for 15 s. All gene-specific primers (Table 1) were synthesized commercially (Sangon Biotech), with Gapdh employed as the endogenous reference. All assays were conducted in technical triplicates.

**TABLE 1:** Sequences of primers used in this study.

Primer	Sequence (5′-3′)
Fos-F	GTT​TCA​ACG​CCG​ACT​ACG​A
Fos-R	CAG​AAC​ATT​CAG​ACC​ACC​TC
Fosb-F	CCG​AGT​CTC​AGT​ACC​TGT​CTT
Fosb-R	CCG​CTC​CAG​TCT​TAT​GAA​TGA​AAC​C
Jun-F	GGG​CAC​ATC​ACC​ACT​ACA​C
Jun-R	AGT​CCT​TAT​CCA​GCC​TGA​GCT​CAA​C
Nr1i2-F	TTTTGGCAGGTCTAATGG
Nr1i2-R	CCA​TGT​TGA​CCC​CAA​ACA​CCA​CCC​C
Prdm1-F	GGTGGTTTATCCTATCCG
Prdm1-R	GCG​AGC​GAC​TGA​CTA​CTC​TTC​CCC​C
Egr1-F	GGA​ACC​GCC​ACT​GCC​AAA​GCG​CCC​A
Egr1-R	CTA​GAC​CAT​CAA​GTT​GGC​ATA​A
Gapdh-F	AGG​TCG​GTG​TGA​ACG​GAT​TTG
Gapdh-R	GGG​GTC​GTT​GAT​GGC​AAC​A

### 2.9 Statistical analysis

Relative gene expression levels were quantified employing the 2^–△△Ct^ method (Livak and Schmittgen, 2001). All statistical analyses and graphical representations were executed utilizing GraphPad Prism 8 software. Quantitative data are presented as the mean ± standard deviation (SD) from at least three independent biological replicates (n = 3). Statistical significance for multiple comparisons against a single control group was evaluated using a one-way analysis of variance (ANOVA) followed by Dunnett’s *post hoc* test. For experiments involving two independent variables, a two-way ANOVA was utilized. For comparisons strictly between two independent groups, an unpaired Student’s t-test was applied. Statistical significance was indicated as follows: ns, *P* > 0.05; *, *P* < 0.05; **, *P* < 0.01; ***, *P* < 0.001; and ****, *P* < 0.0001.

## 3 Results

### 3.1 Assessment of cell viability and clonogenic survival following melittin intervention

Bulleted lists look like this:To characterize the cytotoxic effect of melittin, we first evaluated U14 cell viability using the CCK-8 assay. Quantitative absorbance (OD 450) measurements via a microplate reader revealed a dose-dependent reduction in viability following melittin exposure. Notably, while the 2 μg/mL melittin treatment showed no significant inhibitory effect compared to the control, concentrations at or above 4 μg/mL induced a statistically significant decrease in cell viability (Figure 1A), from which the IC50 value was calculated (Figure 1B).

**FIGURE 1:**
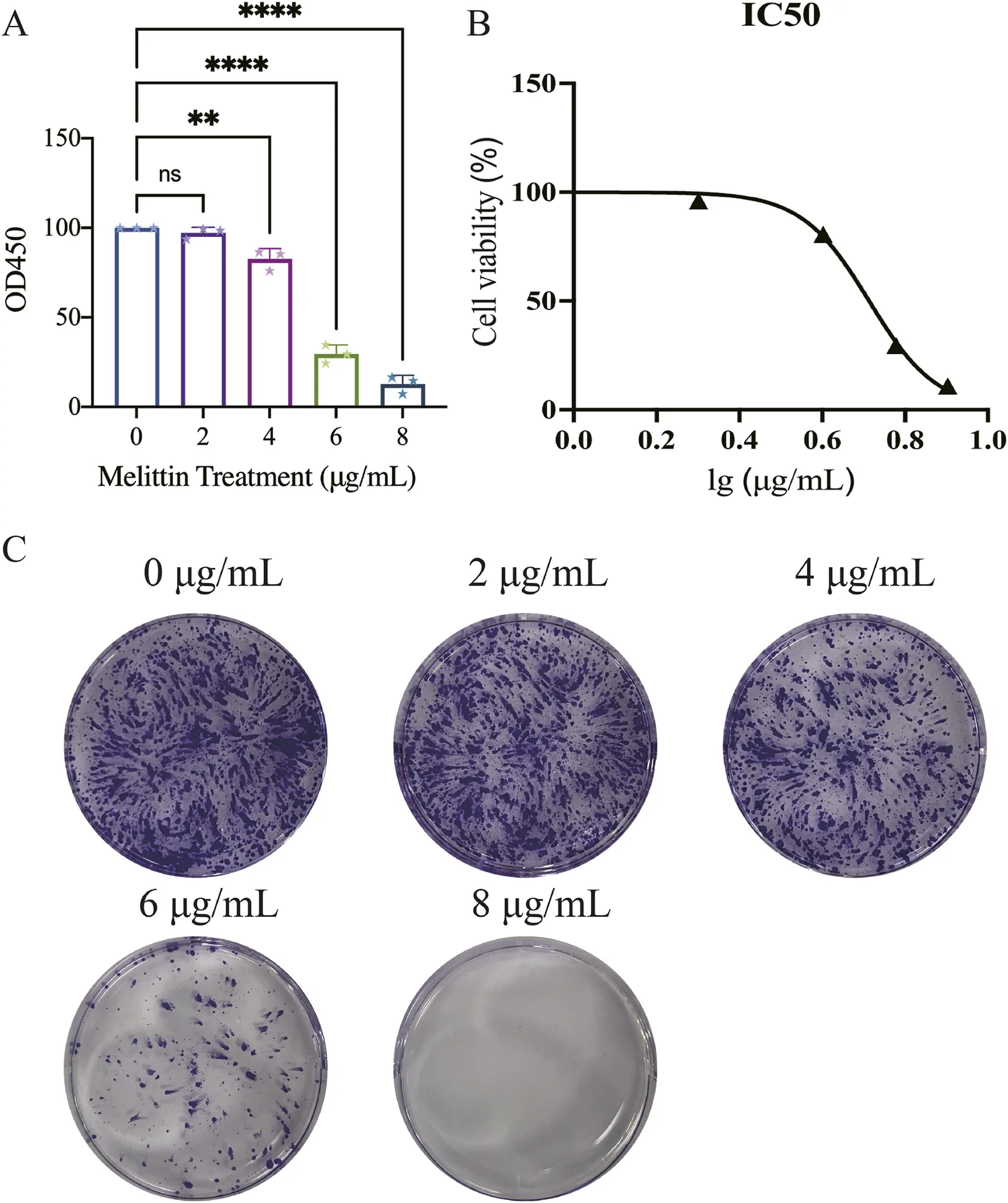
Melittin suppresses the short-term viability and long-term clonogenic survival of murine U14 cervical cancer cells. **(A)** Viability of cells treated with the indicated concentrations of melittin (0, 2, 4, 6, and 8 μg/mL) assessed via Cell Counting Kit-8 (CCK-8) assay. **(B)** Dose-response curve for determining the half-maximal inhibitory concentration (IC50) of melittin. **(C)** The colony formation assay. Data are expressed as mean ± SD from three independent biological replicates (n = 3). Statistical significance was determined using one-way ANOVA followed by Dunnett’s *post hoc* test: ns, not significant (*P* > 0.05), **P < 0.01, ****P < 0.0001 compared to the un-treated control group (0 μg/mL).

Given that acute cytotoxicity assays only define immediate lethality, a colony formation assay was performed to ascertain the impact of melittin on the long-term proliferative potential of U14 cells. Melittin drastically suppressed the clonogenic capacity in a dose-dependent manner (Figure 1C). Control cultures (0 μg/mL) proliferated robustly, forming dense and macroscopic colonies. While the 2 μg/mL treatment largely retained colony formation, exposure to 4 μg/mL noticeably impaired the clonogenic capacity. This inhibitory effect progressed severely at 6 μg/mL, resulting in drastically reduced colony numbers and sizes. Strikingly, intervention at 8 μg/mL led to the complete eradication of clonogenic potential. Collectively, these phenotypic data robustly substantiate the potent anti-cervical cancer activity of melittin. Based on these viability and clonogenic survival analyses, 4 μg/mL was rigorously selected as the optimal intervention concentration for subsequent global transcriptomic investigations.

### 3.2 Assessment of cell migration following melittin intervention

To evaluate the effect of melittin on U14 cell migration, a wound scratch assay was performed. As depicted in Figure 2, the control group exhibited substantial cellular migration into the denuded zone, leading to significant wound closure after 16 h. In contrast, melittin intervention markedly impeded the migratory capacity of U14 cells in a dose-dependent manner. While the 2 μg/mL treatment group showed partial wound healing, exposure to higher concentrations (4 and 6 μg/mL) severely restricted cell motility, resulting in noticeably wider remaining scratch areas. Strikingly, at the highest concentration of 8 μg/mL, the scratch width remained virtually unchanged at 16 h compared to the initial 0 h time point, indicating a near-complete arrest of cell migration (Figure 2).

**FIGURE 2:**
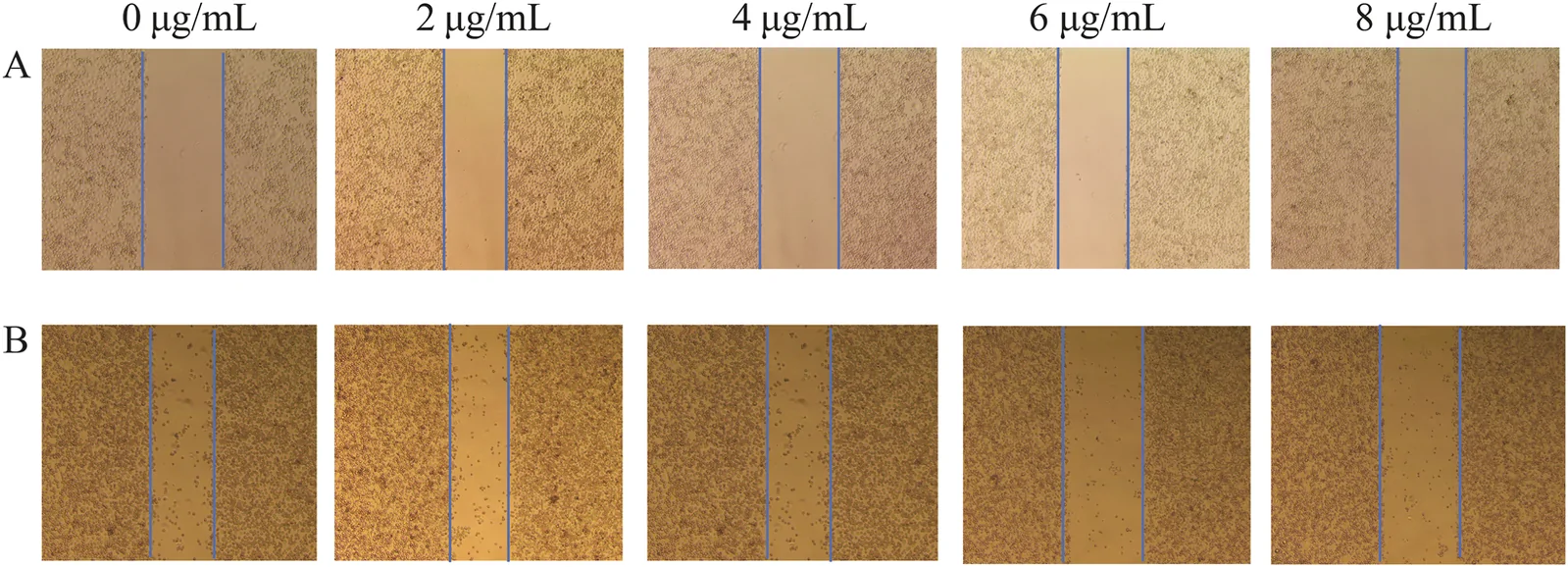
Melittin inhibits the migration of murine U14 cervical cancer cells *in vitro*. Representative images of the wound healing assay at different melittin concentrations (0, 2, 4, 6, and 8 μg/mL) taken at **(A)** 0 h and **(B)** 16 h post-treatment. Images are representative of three independent biological replicates (n = 3).

### 3.3 Assessment of cell apoptosis following melittin intervention

U14 cells treated with varying concentrations of melittin (0, 2, 4, 6, and 8 μg/mL) were analyzed utilizing Annexin V-FITC/PI double staining and flow cytometry. As illustrated in Figure 3, control cells (0 μg/mL) and those exposed to 2 μg/mL melittin exhibited high viability, with the vast majority of cells residing in the live population quadrant (Q1-LL). Furthermore, scanning electron microscopy (SEM) was utilized to detect melittin-induced cell surface ultrastructural alterations; while the untreated control group maintained normal, intact morphology (Figure 4A), cells in the 4 μg/mL melittin-treated group exhibited significant surface damage and roughness (Figure 4B). The flow cytometric scatter plots and corresponding quantitative analysis revealed a predominant increase in the Annexin V+/PI + population (Q1-UR quadrant). Specifically, the proportion of these double-positive cells increased to 10.59% at 4 μg/mL, and surged dramatically to 29.29% and 32.01% following exposure to 6 μg/mL and 8 μg/mL melittin, respectively. Throughout this process, the early apoptotic population (Q1-LR) remained relatively low across all concentrations. Given the well-established membranolytic properties of melittin, the exact nature of this substantial Annexin V+/PI + population remains undefined, though it may represent a possible mixture of caspase-dependent cell death and secondary necrosis induced by direct membrane perforation.

**FIGURE 3:**
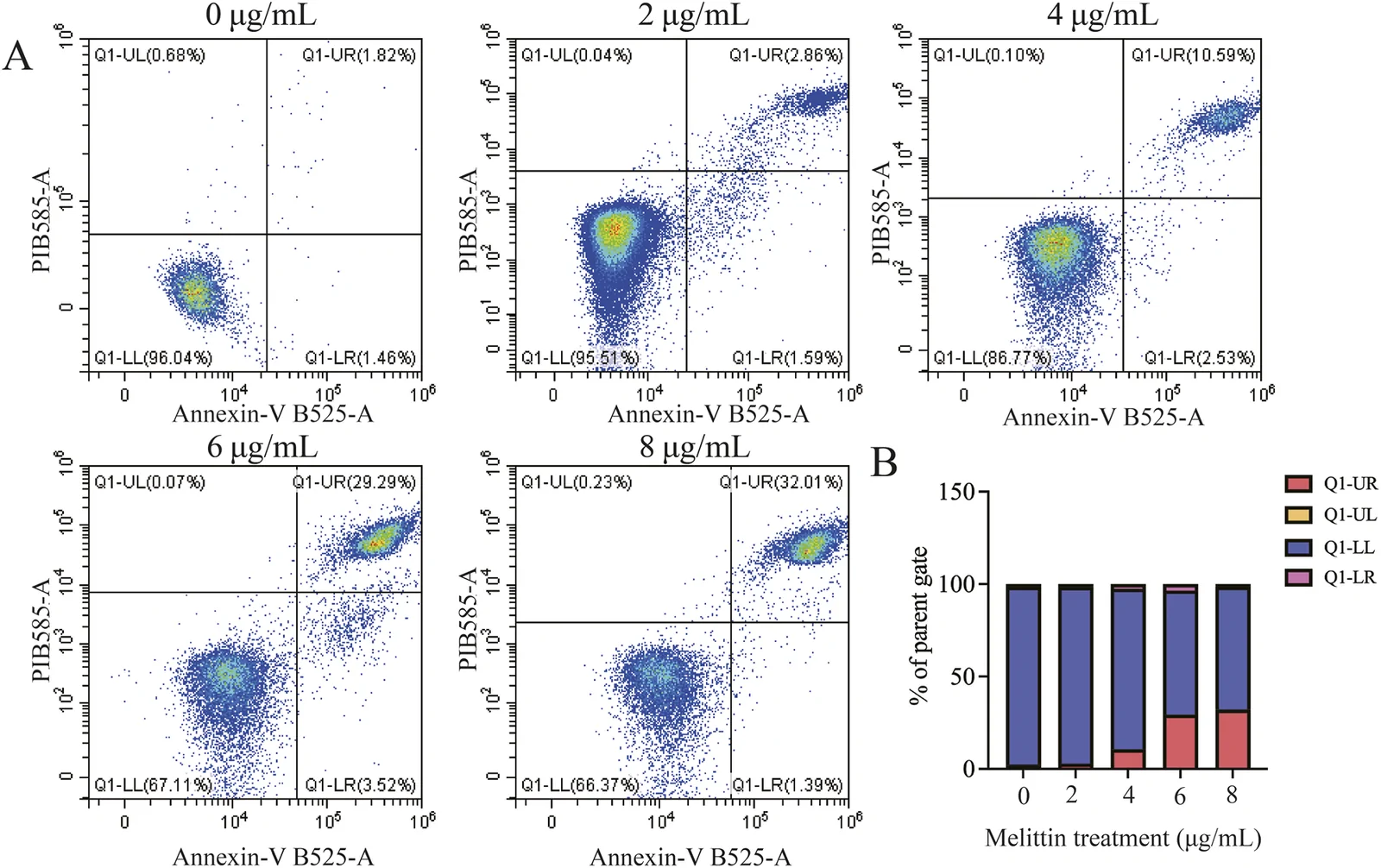
Melittin induces dose-dependent apoptosis in murine U14 cervical cancer cells. **(A)** Representative Annexin V-FITC/PI flow cytometry plots following melittin treatment (0, 2, 4, 6, 8 μg/mL). Quadrants denote necrotic (UL), late apoptotic (UR), live (LL), and early apoptotic (LR) populations. **(B)** Quantitative percentages of the respective cell sub-populations from three independent biological replicates. Data are expressed as mean ± SD from three independent biological replicates (n = 3). Statistical significance for relevant comparisons was evaluated using one-way ANOVA followed by Dunnett’s *post hoc* test.

**FIGURE 4:**
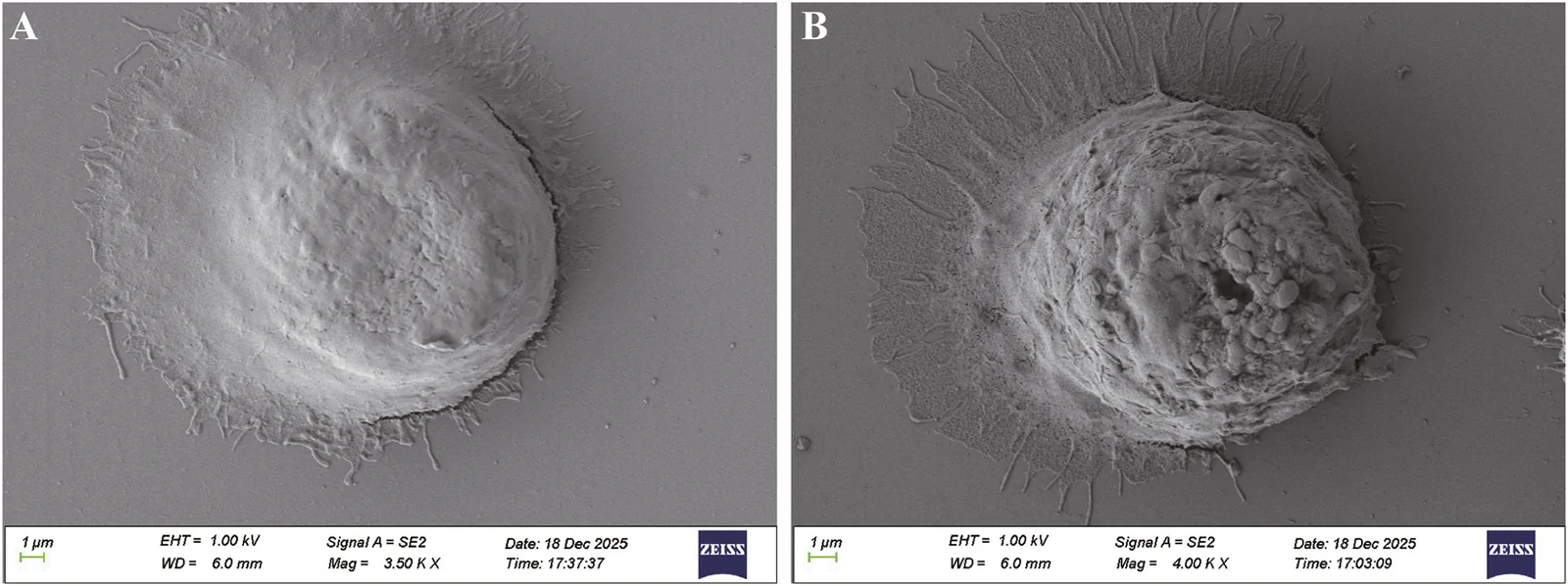
Detection of melittin-induced cell surface ultrastructural alterations in murine U14 cervical cancer cells under scanning electron microscopy (SEM). Representative SEM images of **(A)** the un-treated control group and **(B)** the melittin-treated group at 4 μg/mL. Images are representative of three independent biological replicates (n = 3).

### 3.4 Observation of cellular morphology and ultrastructure following melittin intervention

By using high-resolution SEM, a distinct morphological contrast between the vehicle-treated and melittin-treated cells was onserved (Figure 5). Control cells (Figure 5A) exhibited an intact membrane architecture and normal surface topography. In contrast, following melittin exposure, the treated cells exhibited altered topographical features, characterized by prominent membrane blebbing, surface granulation, structural collapse, and extensive membrane perforations (Figure 5B).

**FIGURE 5:**
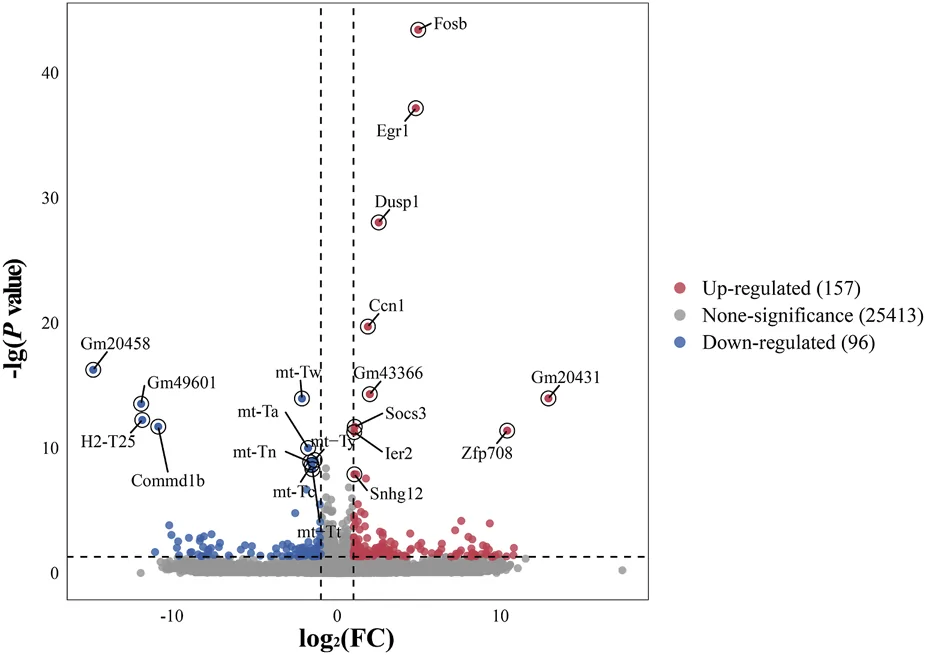
Volcano plot of differentially expressed genes (DEGs) in murine U14 cervical cancer cells following melittin treatment. The x-axis represents the log_2_ fold change (log_2_FC) in gene expression, and the y-axis represents the statistical significance plotted as -lg of the *Q*-value (BH-adjusted *P* value). Each dot corresponds to an individual gene. Red and blue dots denote significantly up- and downregulated genes (|log_2_FC| ≥ 1 and *Q* < 0.05), respectively, while gray dots denote genes without significant difference. The vertical dashed lines indicate the threshold of |log_2_FC| = 1, and the horizontal dashed line indicates the significance threshold of *Q* = 0.05.

### 3.5 Differential expression pattern of genes in melittin-treated U14 cells

Comparative analysis identified a total of 254 DEGs between the melittin-treated and control groups, consisting of 158 upregulated and 96 downregulated genes. Specifically, *Fos* (log_2_FC = 6.10, *Q* < 0.001), *Fosb* (log_2_FC = 4.96, *Q* < 0.001), and *Egr1* (log_2_FC = 4.81, *Q* < 0.001) emerged as the top three upregulated genes. Conversely, the most pronounced downregulation was observed in the uncharacterized genomic loci *Gm20458* (log_2_FC = −14.94) and *Gm49601* (log_2_FC = −12.01), as well as the mitochondrial tRNA gene *mt-Tw* (log_2_FC = −2.16, *Q* < 0.001) (Figure 3).

### 3.6 Functional and pathway enrichment analyses of DEGs

GO analysis identified significant enrichment in biological process (BP) terms related to RNA polymerase II transcriptional regulation, signal transduction, differentiation, immunity, and apoptotic regulation (Figure 6; Supplementary Table S1). Other enriched BP terms included GPCR signaling and responses to xenobiotics, IL-6, and IL-17. Cellular component (CC) mapping localized these DEGs to the membrane, nucleus, mitochondrion, endoplasmic reticulum, and Golgi apparatus, while molecular function (MF) was dominated by DNA-binding transcription factor activities (Figure 6A).

**FIGURE 6:**
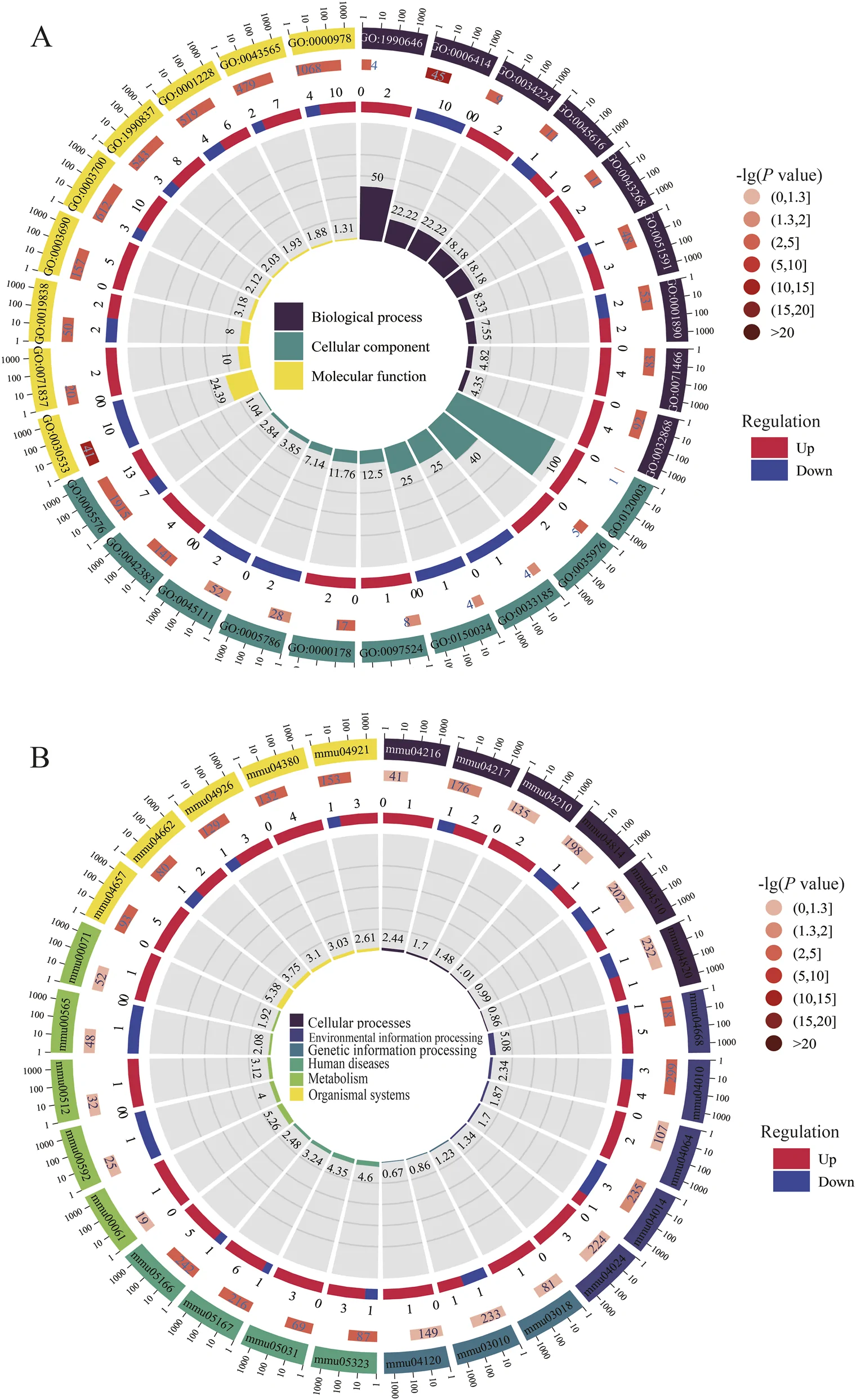
Circos plots of Gene Ontology (GO) terms **(A)** and Kyoto Encyclopedia of Genes and Genomes (KEGG) pathways **(B)** enriched by DEGs in murine U14 cervical cancer cells following melittin treatment.

KEGG profiling linked DEGs predominantly to signaling pathways involved in cellular stress responses and immune regulation, enriching in the TNF, IL-17, and MAPK pathways. Gene-level mapping highlighted the significant upregulation of immediate-early genes (IEGs) (*Fos*, *Fosb*, *Jun*, and *Egr2*). *Fos* and *Fosb* were mapped to the enriched Activated Protein 1 (AP-1) complex, while Jun was involved in MAPK/TNF-driven proliferative and inflammatory cascades. *Egr2* was linked to sequence-specific DNA binding. Additionally, the ciliary-associated gene *Tulp2* was upregulated, whereas the angiogenic regulator *Etv2* was markedly downregulated (Figure 6B).

### 3.7 Reactome pathway enrichment analysis

To systematically decode the transcriptomic alterations at the pathway level, Reactome enrichment analysis was conducted, yielding 20 significantly enriched biological cascades (Figure 7). The functional landscape revealed a pronounced divergence between up- and downregulated gene modules. Notably, the Formation of AP-1 complex pathway displayed the most profound statistical significance (*P* = 0.0039, *Q* = 0.02, Rich factor ≈0.95), driven exclusively by upregulated genes. Additional cascades dominated by upregulated genes encompassed specific metabolic and receptor adaptations, such as Myoglobin binds oxygen and CHRM4 binds acetylcholine.

**FIGURE 7:**
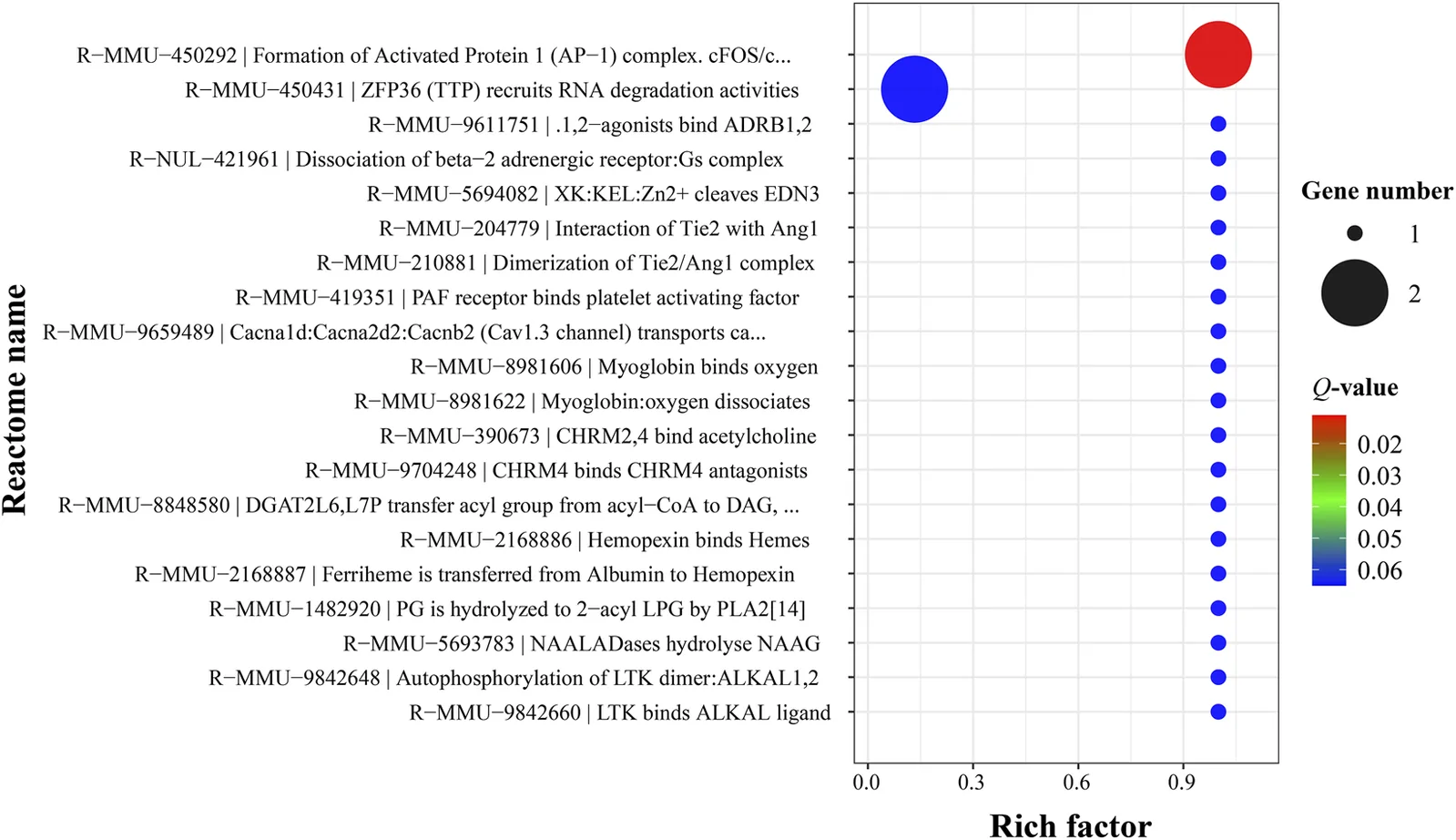
Bubble plot of Reactome pathways enriched by DEGs in melittin-treated murine U14 cervical cancer cells. The Rich factor represents the ratio of DEGs annotated to a specific pathway to the total number of background genes in that same pathway.

Conversely, a distinct functional module characterized by downregulated genes was predominantly mapped to RNA turnover and angiogenesis-related signaling. This included significant enrichment of ZFP36 (TTP) recruits RNA degradation activities alongside vascular development networks, which include modules associated with the angiopoietin-Tie2 cascade. Additionally, cascades involving the autophosphorylation of LTK dimers, beta-adrenergic receptor signaling, and platelet-activating factor receptor signaling also reached statistical significance. Taken together, these data objectively delineate a transcriptomic signature defined by the induction of AP-1-associated networks and the concurrent suppression of broader angiogenesis-related networks (Figure 7).

### 3.8 GSEA of DEGs

Global transcriptomic profiling via GSEA demonstrated a significant suppression of translation-related gene sets following melittin treatment. The most negatively enriched GO terms were structural constituent of ribosome (NES = −3.16, FDR <0.001) and translational elongation (NES = −3.15, FDR <0.001), alongside triplet codon-amino acid adaptor activity (NES = −3.07, FDR <0.001) and cytoplasmic translation (NES = −2.95, FDR <0.001) (Figure 8).

**FIGURE 8:**
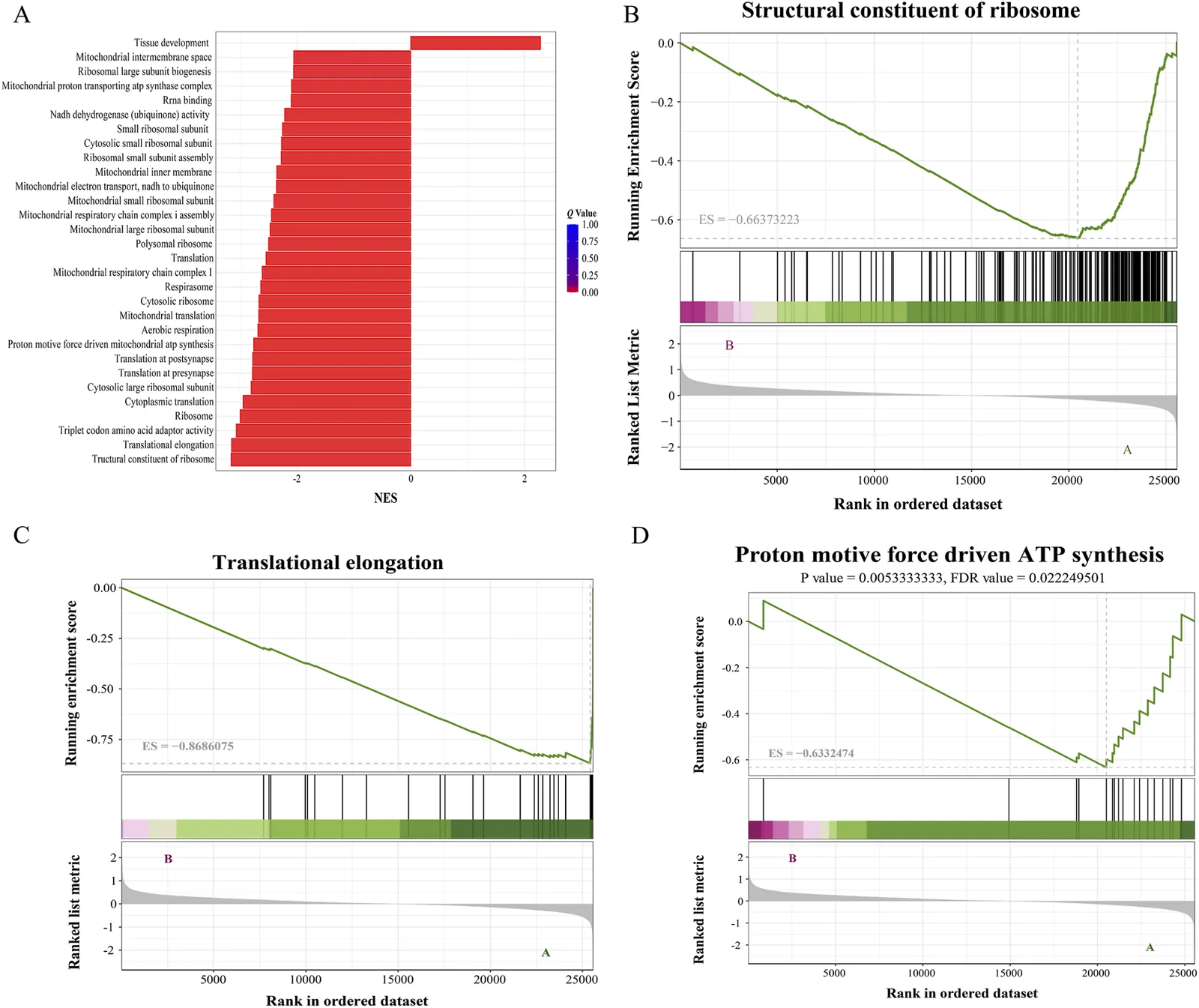
Gene set enrichment analysis (GSEA) of translation- and energy metabolism-related gene sets in cervical cancer cells following melittin treatment. **(A)** Bar chart of negatively and significantly enriched GO terms. **(B–D)** Representative enrichment plots of core downregulated functional gene sets associated with structural constituent of ribosome, translational elongation, and proton motive force-driven mitochondrial ATP synthesis.

Consistent with the GO analysis, KEGG pathway GSEA identified Ribosome (NES = −3.34, FDR <0.001). This was closely followed by a substantial reduction in the Oxidative phosphorylation pathway (NES = −2.42, FDR <0.001). Furthermore, significant negative enrichment was observed in networks controlling proteasomal degradation and those associated with neurodegenerative diseases (NES from −1.62 to −1.88, FDR <0.05) (Figure 9).

**FIGURE 9:**
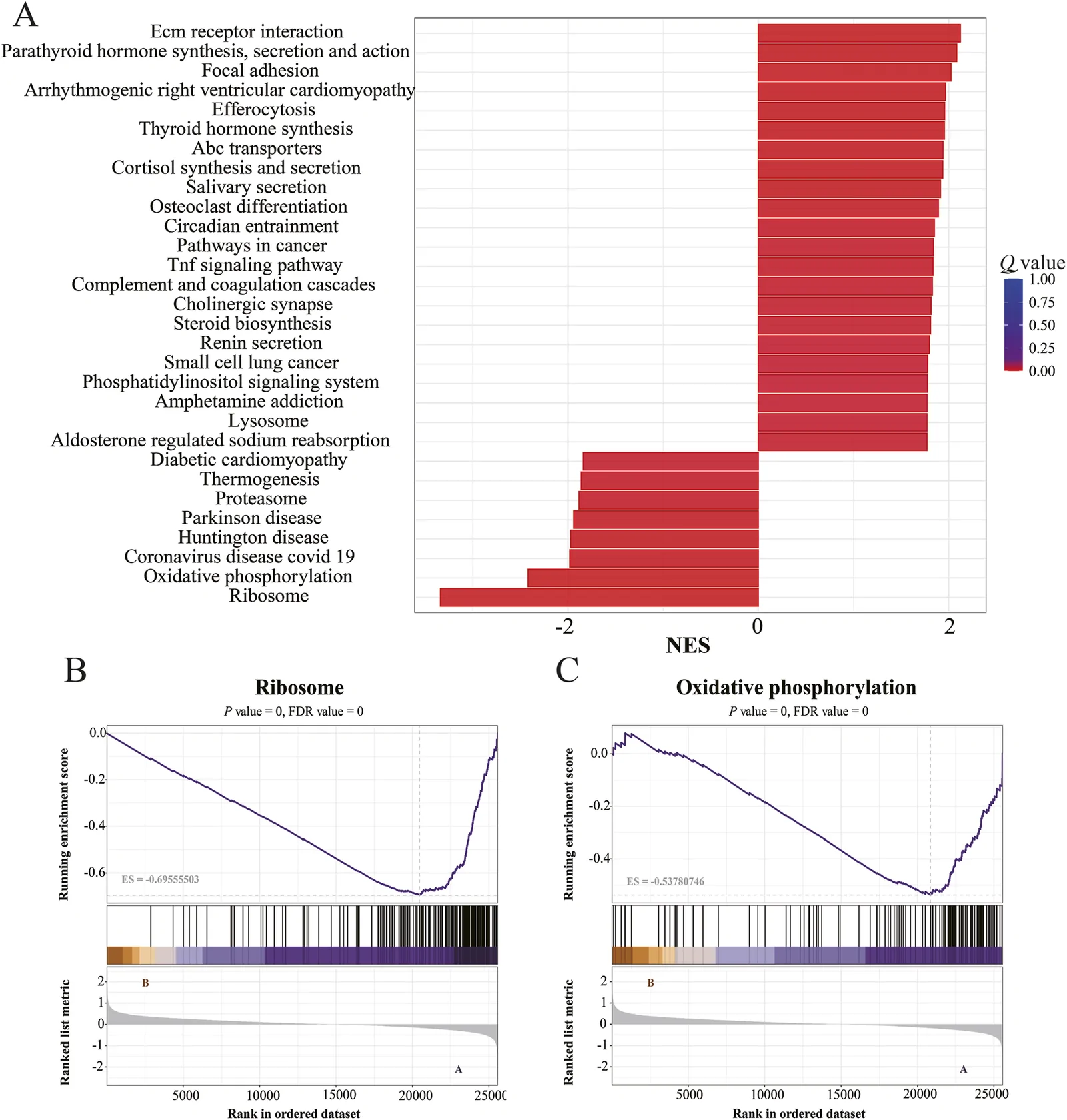
KEGG pathway-based GSEA uncovers the repression of core translational and metabolic pathways in melittin-treated cervical cancer cells **(A)** Horizontal bar plot of enriched KEGG pathways **(B–C)** Representative GSEA enrichment plots for the two core downregulated pathways: the ribosome pathway and the oxidative phosphorylation pathway.

### 3.9 PPI network analysis of DEGs

Topological evaluation of the PPI network delineated a highly centralized and densely interconnected regulatory module. Only interactions with a score >0.400 are displayed. Within this network hierarchy, Jun emerged as the principal hub node, exhibiting the highest degree of connectivity (degree = 22). Furthermore, a distinct sub-network of immediate interacting partners, predominantly consisting of Fos, Fosb, Egr1, Dusp1, Ccn1, and Zfp36, densely aggregated directly around this central Jun anchor (Figure 10).

**FIGURE 10:**
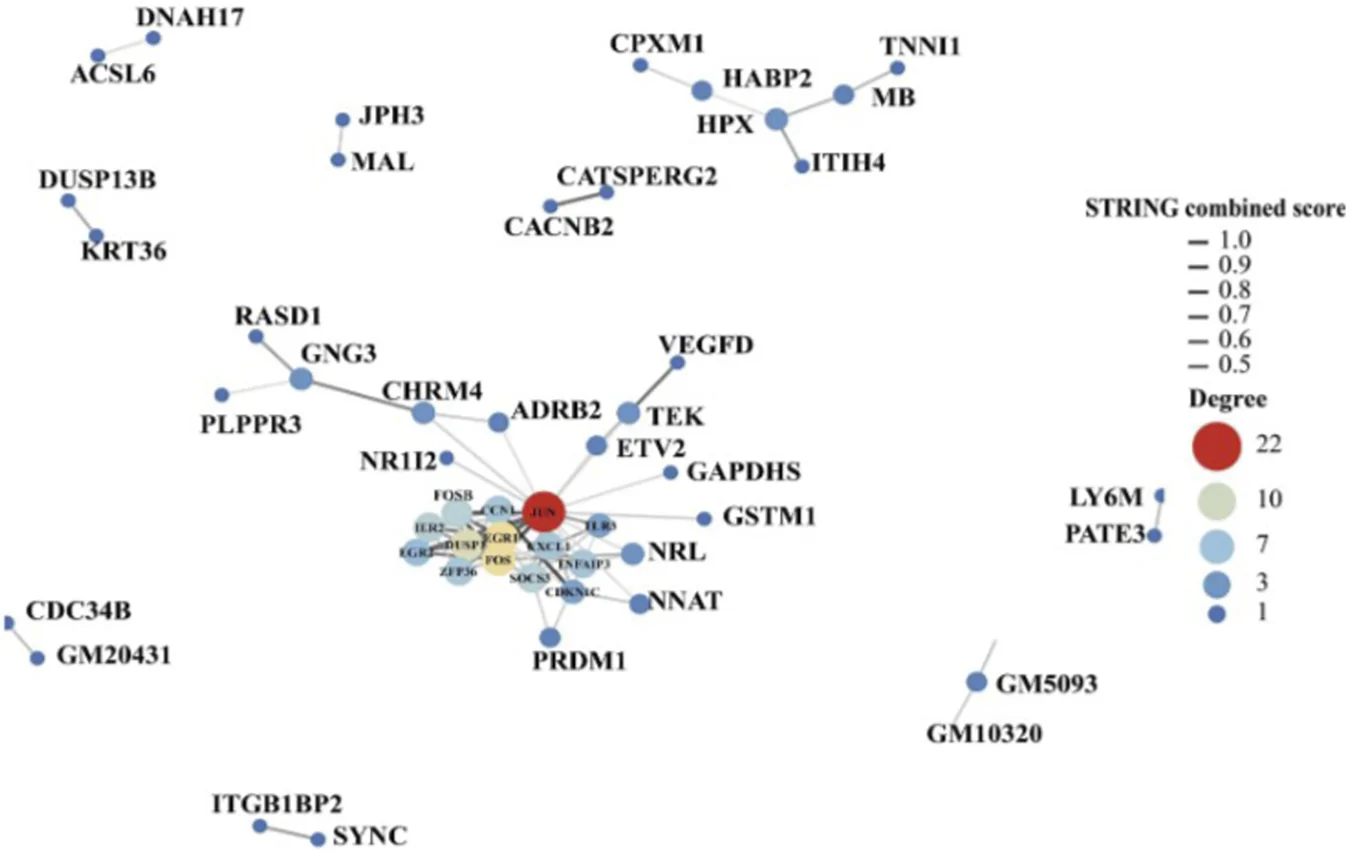
Protein-protein interaction (PPI) network of DEGs in melittin-treated murine U14 cervical cancer cells. A minimum required interaction score of >0.400 (medium confidence) was set as the threshold for the STRING database analysis.

### 3.10 RT-qPCR validation of transcriptome data

RT-qPCR assay showed a profound, highly significant transcriptional burst of *Fos* (*P* < 0.0001), accompanied by a significant concurrent induction of *Fosb* (*P* < 0.05). Concurrently, although *Egr1*, *Jun*, and *Prdm1* displayed a trend toward upregulation and *Nr1i2* trended downregulation, these alterations did not reach statistical significance. The results verified the reliability of transcriptome data used in this study (Figure 11).

**FIGURE 11:**
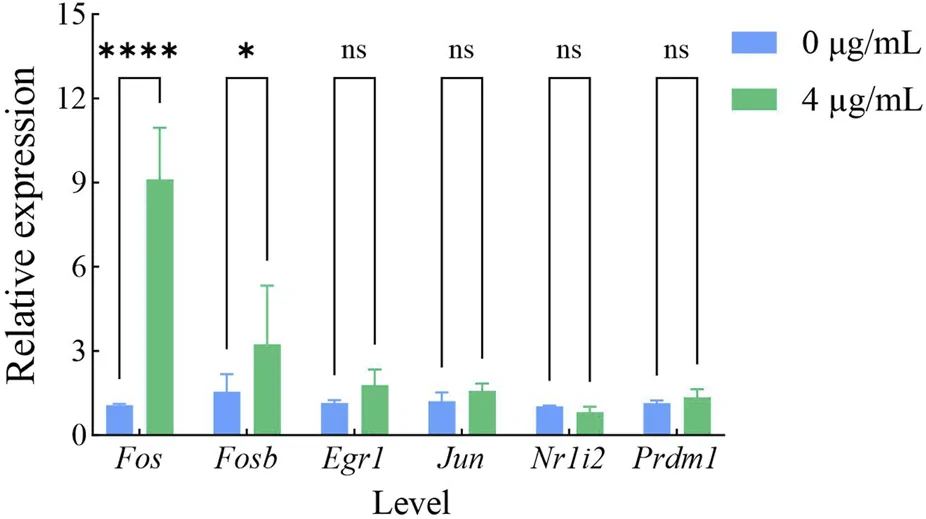
Relative mRNA expression levels of six target genes under 4 μg/mL melittin treatment assessed by RT-qPCR. Data are expressed as mean ± SD from three independent biological replicates (n = 3). Statistical significance was determined using Student’s t-test: ns, not significant (*P* > 0.05). *P < 0.05, ****P < 0.0001.

## 4 Discussion

Cervical cancer represents one of the most prevalent gynecological malignancies worldwide, and the clinical limitations of conventional chemoradiotherapy highlight the urgent need for novel, highly effective therapeutic agents (Vaghasia et al., 2025). In recent years, natural antimicrobial peptides have garnered significant attention in oncology. Melittin, the principal active component of honeybee venom, has emerged as a particularly potent candidate due to its renowned membrane-disrupting properties and broad-spectrum anti-tumor activities (Zhang et al., 2024; Duffy et al., 2020). Despite its well-documented cytotoxicity across various solid tumors, the profound intracellular molecular perturbations and the global transcriptomic reprogramming induced by melittin in cervical cancer remain inadequately elucidated (Rady et al., 2017; Lv et al., 2023; Kong et al., 2016). To address this knowledge gap, the present study comprehensively investigated the anti-cancer efficacy of melittin in murine U14 cervical cancer cells by integrating macroscopic phenotypic characterizations with in-depth transcriptomic profiling.

Phenotypically, melittin exhibited potent, dose-dependent cytotoxicity against U14 cells, profoundly suppressing both acute viability and long-term clonogenic survival (Figure 1). Flow cytometry confirmed that this lethal effect was characterized by a rapid increase in the Annexin V+/PI + population. Given the membranolytic nature of melittin, this population may represent a possible mixture of caspase-dependent cell death and secondary necrosis induced by direct membrane perforation (Figure 4). Additional experiment is needed to uncover the underlying manner. Since caspase-3/7 assays and apoptosis inhibitors were not utilized in this study, the exact nature of this cell death remains undefined and warrants future biochemical investigation.

Ultrastructural analysis via SEM provided direct visual evidence of this process, revealing severe membrane blebbing, structural collapse, and extensive perforation. These physical disruptions are consistent with melittin’s amphipathic nature, which facilitates its rapid insertion into lipid bilayers to execute membrane lysis (Zarrinnahad et al., 2018; Ye et al., 2021) (Figure 5). Furthermore, higher concentrations of melittin virtually abolished cell motility in wound scratch assays. This robust anti-migratory effect aligns with recent pharmacological findings across various solid tumors (Jeong et al., 2014; Salimian et al., 2022; Jo et al., 2012), confirming melittin’s capacity to restrict cervical cancer progression (Figure 2).

To elucidate the underlying molecular alterations, GO enrichment analysis provided initial insights into the global transcriptomic shift. The predominant enrichment in biological processes governing RNA polymerase II-mediated transcription, signal transduction, and apoptotic regulation indicates that the physical disturbance caused by melittin rapidly translates into a systemic nuclear transcriptional response (Figure 6A). Following this, KEGG pathway topological profiling highlighted significant enrichment in environmental information processing networks, notably the TNF, IL-17, and MAPK signaling pathways. Although the acute treatment duration (20 min) yielded a relatively focused set of 254 DEGs, this specific timeframe is uniquely optimal for capturing the sharp induction of immediate-early genes (IEGs) before secondary downstream cascades obscure the initial regulatory network. Gene-level mapping within these pathways revealed a striking upregulation of IEGs, including *Fos*, *Fosb*, *Jun*, and *Egr2*. While MAPK and TNF signaling cascades are traditionally associated with cell proliferation under physiological conditions (Figure 6B), recent transcriptomic insights reveal that the acute hyperactivation of IEGs under severe external intervention often signifies an unresolvable cellular stress response that drives apoptosis rather than survival (Yue and López, 2020; Xue et al., 2025). Concurrently, the marked downregulation of *Etv2*, a key regulator enriched in angiogenesis, suggests that melittin simultaneously suppresses pro-angiogenic signals crucial for tumor progression.

To further dissect these transcriptomic shifts, Reactome enrichment analysis was employed, uncovering a distinct divergence in pathway functionality. On one hand, melittin overwhelmingly triggered the AP-1 complex formation pathway, suggesting the AP-1 transcription factor complex as the primary regulatory hub governing the cellular stress response. On the other hand, it precipitated a significant decline in modules related to vascular development, highlighted by the downregulation of the transcription factor *Etv2*. While *Etv2* is not a direct component of the Tie2/Ang1 signaling cascade, it is a critical upstream regulator of endothelial development and angiogenesis (Saharinen et al., 2017; Eklund et al., 2017). Therefore, the profound transcriptional suppression of *Etv2* and its associated networks corroborates the anti-migratory phenotype observed in our cellular assays, pointing to a potential disruption in essential tumor-stroma crosstalk (Figure 7).

To overcome the threshold-dependent biases of conventional DEG profiling, we utilized GSEA to capture system-wide functional shifts. GSEA revealed a sweeping, highly significant suppression of translation- and energy metabolism-related gene sets. Pathways governing ribosomal biogenesis and mitochondrial oxidative phosphorylation exhibited profound negative enrichment. Rapidly proliferating cancer cells maintain an absolute biosynthetic and energetic dependency on hyperactive translation and oxidative ATP production (Pelletier et al., 2015; Ashton et al., 2018) (Figures 8, 9). Emerging cancer therapeutic strategies heavily emphasize targeting these exact vulnerabilities (Vasan et al., 2020). The global collapse of ribosomal architecture and oxidative phosphorylation indicates that melittin severely impairs the translational and energetic capacities of U14 cells, depriving them of the metabolic pillars required for survival.

Further, topological evaluation of the PPI network delineated a highly centralized regulatory interactome, structurally reinforcing our functional enrichment findings. Anchored by Jun as the primary topological hub, this network features a dense sub-cluster containing Fos, Fosb, Egr1, Dusp1, Ccn1, and Zfp36 (Figure 10). Crucially, our subsequent RT-qPCR quantitative validation further refined this regulatory landscape. While global RNA-seq identified a broad engagement of the early response network, targeted RT-qPCR analysis revealed that this acute stress response was fundamentally driven by a profound and highly significant transcriptional burst specific to *Fos* and *Fosb* (Figure 11). In contrast, alterations in other network nodes (like *Jun*, *Egr1*, and *Prdm1*) exhibited non-significant trends, underscoring *Fos* and *Fosb* as the principal responsive targets under this specific melittin treatment regimen. Although *Jun* emerged as a central hub in the PPI network, its mRNA levels remained relatively stable. This is biologically plausible, as AP-1 predominantly functions as a *Jun-Fos* heterodimer. *Jun* is constitutively expressed and its activity is largely dictated by rapid post-translational modifications (such as phosphorylation) rather than *de novo* transcription. Therefore, the dramatic transcriptional spike in *Fos* and *Fosb* is sufficient to shift the stoichiometric balance, driving AP-1 activation by pairing with the pre-existing intracellular pool of *Jun* proteins.

The profound upregulation of *Fos* and *Fosb*, coupled with massive apoptotic events, highlight a pivotal role of the AP-1 complex in melittin-induced cytotoxicity. However, it is important to note that AP-1 signaling exerts dual, context-dependent roles, acting as both a driver of apoptosis and a mediator of cell survival (Qiao et al., 2021; Bejjani et al., 2019). Under the severe membranolytic stress induced by melittin, the robust transcriptional induction of AP-1 likely reflects an overwhelmed cellular stress-response mechanism rather than an exclusive pro-apoptotic cascade. Rather than operating through a strictly linear mechanism, AP-1 modulates cell fate via intricate crosstalk with alternative signaling cascades. Within the context of our findings, the concurrent AP-1 transcriptional burst, ribosomal suppression, and *Etv2*-mediated angiogenic suppression act synergistically to drive the cellular fate toward apoptosis. Future functional decoupling studies utilizing specific gene knockdown are necessary to definitively confirm the pro-apoptotic causality of AP-1 in this model.

In summary, melittin effectively suppresses murine U14 cervical cancer cells through a highly synchronized, multi-targeted mechanism. The robust anti-tumor efficacy is driven by direct plasma membrane lysis, followed by a sweeping transcriptomic collapse of ribosomal biogenesis and oxidative metabolism, the downregulation of angiogenesis-related transcription factors (e.g., *Etv2*), and the marked induction of the AP-1 transcriptional stress network. Collectively, these molecular insights provide an exploratory molecular framework for evaluating melittin as a potential therapeutic candidate for cervical carcinoma.

Despite the transcriptomic and phenotypic evidence presented, we acknowledge certain limitations that warrant future investigation. Firstly, although prior studies have established melittin’s selective cytotoxicity against cervical cancer (Kim et al., 2015), this current work lacks a parallel non-tumorigenic murine cell control, precluding a direct calculation of the therapeutic index. Therefore, these findings serve as an exploratory transcriptomic framework rather than definitive mechanistic proof. Secondly, as noted earlier, without the use of specific apoptosis inhibitors or caspase-3/7 assays, the exact nature of the cell death mechanism cannot be definitively confirmed. Thirdly, while the suppression of ribosomal biogenesis and oxidative phosphorylation presents a compelling metabolic signature, it lacks direct functional validation (e.g., ATP quantification or puromycin-based translation assays). Ultimately, while the transcriptomic induction of the AP-1 network is robust, the execution of this pathway relies on protein-level dynamics. Consequently, ensuing studies must prioritize Western blot validation of Fos, Fosb, and Jun, alongside luciferase reporter assays to confirm AP-1 transcriptional activity. Future investigations bridging *in vivo* toxicity assessments and functional protein dynamics are necessary to fully substantiate melittin’s clinical potential.

## 5 Conclusion

In conclusion, this study systematically validates the anti-cervical cancer efficacy of melittin through a dual-level investigation. Experimentally, melittin exerts potent cytotoxicity by directly disrupting the plasma membrane and effectively abolishing cell migration and survival. Mechanistically, downstream transcriptomic profiling and RT-qPCR validation reveal that this physical damage initiates a massive intracellular crisis, highlighted by the profound suppression of ribosomal translation and oxidative metabolism, the suppression of *Etv2*-associated angiogenic networks, and the profound transcriptomic induction of a *Fos/Fosb*-dominated AP-1 stress interactome. This multi-modal mechanism, combining structural, metabolic, and transcriptional targeting, offers an exploratory molecular framework and rationale for future experimental validation of melittin as a potential therapeutic agent in the treatment of cervical carcinoma.

## Data Availability

The original contributions presented in the study are included in the article and its Supplementary Material. Specifically, the full datasets generated from the transcriptomic analysis, including complete lists of DEGs and full functional enrichment results, are provided as Supplementary Table S1 and Supplementary Table S2. Furthermore, the full raw RNA-seq dataset supporting the conclusions of this article has been deposited in the National Genomics Data Center (NGDC) BioProject database and can be accessed directly via the permanent link: https://ngdc.cncb.ac.cn/bioproject/browse/PRJCA068439.

## Glossary

AP-1: Activator Protein 1
BP: Biological Process
CC: Cellular Component
CCK-8: Cell Counting Kit-8
DEGs: Differentially Expressed Genes
FC: Fold Change
FDR: False Discovery Rate
FITC: Fluorescein Isothiocyanate
GO: Gene Ontology
GPCR: G Protein-Coupled Receptor
GSEA: Gene Set Enrichment Analysis
HPV: Human Papillomavirus.
IC50: Half-Maximal Inhibitory Concentration
IEGs: Immediate-Early Genes.
IL-6: Interleukin-6
IL-17: Interleukin-17
KEGG: Kyoto Encyclopedia of Genes and Genomes
MAPK: Mitogen-Activated Protein Kinase
MF: Molecular Function
NES: Normalized Enrichment Score
OD: Optical Density
PBS: Phosphate-Buffered Saline
PD-1: Programmed Death Protein 1
PD-L1: Programmed Death-Ligand 1
PI: Propidium Iodide
PPI: Protein-Protein Interaction
qRT-PCR: Quantitative Real-Time Polymerase Chain Reaction
RNA-seq: RNA Sequencing
SD: Standard Deviation
SEM: Scanning Electron Microscopy
Tie2: TEK Receptor Tyrosine Kinase
TNF: Tumor Necrosis Factor
TTP: Tristetraprolin (Zinc Finger Protein 36, ZFP36)
