# Supplementary material for: Exploratory transcriptomic profiling of melittin’s efficacy in the treatment of murine cervical cancer cells
Source: Front Cell Dev Biol. 2026 Oct 14;14:1975665. doi: 10.3389/fcell.2026.1975665 (PMC13713771; doi:10.3389/fcell.2026.1975665)
Supplement: Supplementary file 1 [file Table1.docx]

Supplementary Material

Table S1. GO term and KEGG pathway enrichment analyses of the differentially expressed genes in melittin-treated U14 cells.

| **Category** | **Term/Pathway** | ***P* value** | ***Q* value** | **Nmuber** |
| --- | --- | --- | --- | --- |
| BP | Regulation of transcription by RNA polymerase II | 0.004 | 0.089 | 16 |
| BP | Cellular response to xenobiotic stimulus | 0.001 | 0.089 | 4 |
| BP | Cellular response to interleukin-17 | 0.003 | 0.089 | 2 |
| BP | Positive regulation of interleukin-6 production | 0.003 | 0.089 | 4 |
| BP | Cell differentiation | 0.029 | 0.147 | 10 |
| BP | Immune system process | 0.316 | 0.417 | 5 |
| BP | Apoptotic process | 0.455 | 0.537 | 4 |
| BP | G protein-coupled receptor signaling pathway | 0.992 | 0.996 | 5 |
| CC | Membrane | 0.515 | 0.584 | 49 |
| CC | Nucleus | 0.918 | 0.934 | 28, |
| CC | Mitochondrion | 0.078 | 0.205 | 15 |
| CC | Endoplasmic reticulum | 0.120 | 0.252 | 13 |
| CC | Golgi apparatus | 0.618 | 0.672 | 7 |
| MF | DNA-binding transcription factor activity | < 0.001 | 0.014 | 13 |
| KEGG | TNF signaling pathway | <0.001 | 0.002 | 6 |
| KEGG | IL-17 signaling pathway | <0.001 | 0.003 | 5 |
| KEGG | MAPK signaling pathway | <0.001 | 0.013 | 7 |
